# Antibacterial activity of ceramide and ceramide analogs against pathogenic *Neisseria*

**DOI:** 10.1038/s41598-017-18071-w

**Published:** 2017-12-15

**Authors:** Jérôme Becam, Tim Walter, Anne Burgert, Jan Schlegel, Markus Sauer, Jürgen Seibel, Alexandra Schubert-Unkmeir

**Affiliations:** 1 Institute of Hygiene and Microbiology, Julius-Maximilian University Wuerzburg, Wuerzburg, Germany; 2 Institute for Organic Chemistry, Julius-Maximilian University Wuerzburg, Wuerzburg, Germany; 3 Department of Biotechnology and Biophysics, Julius-Maximilian University Wuerzburg, Wuerzburg, Germany

## Abstract

Certain fatty acids and sphingoid bases found at mucosal surfaces are known to have antibacterial activity and are thought to play a more direct role in innate immunity against bacterial infections. Herein, we analysed the antibacterial activity of sphingolipids, including the sphingoid base sphingosine as well as short-chain C_6_ and long-chain C_16_-ceramides and azido-functionalized ceramide analogs against pathogenic *Neisseriae*. Determination of the minimal inhibitory concentration (MIC) and minimal bactericidal concentration (MBC) demonstrated that short-chain ceramides and a ω-azido-functionalized C_6_-ceramide were active against *Neisseria meningitidis* and *N. gonorrhoeae*, whereas they were inactive against *Escherichia coli* and *Staphylococcus aureus*. Kinetic assays showed that killing of *N*. *meningitidis* occurred within 2 h with ω–azido-C_6_-ceramide at 1 X the MIC. Of note, at a bactericidal concentration, ω–azido-C_6_-ceramide had no significant toxic effect on host cells. Moreover, lipid uptake and localization was studied by flow cytometry and confocal laser scanning microscopy (CLSM) and revealed a rapid uptake by bacteria within 5 min. CLSM and super-resolution fluorescence imaging by *direct* stochastic optical reconstruction microscopy demonstrated homogeneous distribution of ceramide analogs in the bacterial membrane. Taken together, these data demonstrate the potent bactericidal activity of sphingosine and synthetic short-chain ceramide analogs against pathogenic *Neisseriae*.

## Introduction

**Figure 1:**
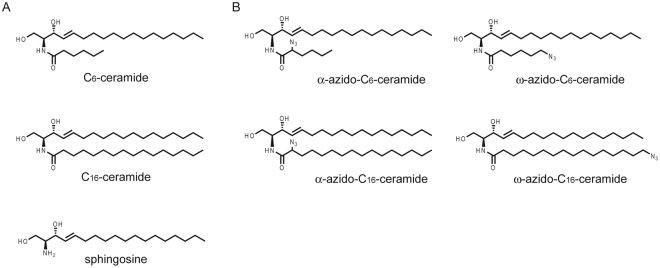
(**A**) Chemical structures of commercial available unmodified short-chain C_6_-ceramide (d18:1/6:0) and long-chain C_16_-ceramides (d18:1/16:0) and d-*erythro*-Sphingosine. (**B**) Chemical structures of α-azido-C_6_-ceramide, ω-azido-C_6_-ceramide, α-azido-C_16_-ceramide and ω-azido-C_16_-ceramide.

Sphingolipids are composed of a structurally related family of backbones termed sphingoid bases, which are sometimes referred to as ‘long-chain bases’ or ‘sphingosines’. The long chain (sphingoid) bases are aliphatic amines, containing two or three hydroxyl groups, and often a distinctive trans-double bond in position 4. The most abundant sphingoid base in animal tissues is sphingosine ((2 *S*,3 *R*,4*E*)-2-amino-4-octadecen-1,3-diol) with a C18 aliphatic chain, hydroxyl groups in positions 1 and 3 and an amine group in position 2 (see also Fig. 1). If the amine group of a sphingoid base is *N*-acylated with a fatty acid moiety, a ceramide molecule is formed. Ceramides consist of a long-chain or sphingoid base linked to a fatty acid via an amide bond. They are formed as the key intermediates in the biosynthesis of all complex sphingolipids. Fatty acids within ceramide molecules may vary in chain length^1^ and are usually saturated, or with a single double bond or an α-hydroxyl group. Natural sphingolipids can be further chemically modified, resulting in derivatives that possess new properties. For example, sphingosine can be coupled with phenethyl isothiocyanatecan (PEITC) which leads to a significant increase of its antitumour activity towards human leukaemia cell growth as compared to sphingosine or PEITC alone^2^.

Interestingly, many of the naturally occurring and synthetic sphingoid bases are cytotoxic for cancer cells and some have been recognized to exert antibacterial activity against pathogenic microorganisms^3–5^. So far, growth inhibitory activity has been shown against Gram-positive and Gram-negative bacteria, fungi and microalgae^6–10^. Interestingly, recent studies demonstrated that the sphingoid base sphingosine is also an important first-line defence of healthy airways against *Pseudomonas aeruginosa* ^11^. Recently, a series of ceramide analogs was synthesized and analysed for the growth inhibitory effect on *Chlamydia trachomatis* ^12^. Moreover, several synthetic dihydrosphingosine analogs have been demonstrated to be active against multi-drug resistant strains of *Mycobacterium tuberculosis* ^13^.

The inhibitory activity of sphingolipids towards microorganisms is thought to be a result of their ability to interact with the microbial cell wall^14^, however the exact mechanism is still not known. As a result the leakage of cellular content and consequently microbial death occurs. Alternatively, lipids may penetrate and accumulate in the cytoplasm and may interfere with cell metabolism.

The present study details the antimicrobial activity of sphingolipids, focussing on sphingosines, ceramides and chemically modified ceramide-analogs against pathogenic *Neisseriae*. As an exclusive human pathogen, *Neisseria meningitidis* colonizes the upper respiratory tract of approximately 10–40% of the healthy population^15,16^. In rare cases, the bacteria can cause devastating invasive infections, resulting in sepsis and meningitis, mostly in young infants and toddlers. *N. gonorrhoeae* is a human pathogen responsible for genital tract infection (gonorrhoea). Occasionally, the organism can disseminate as a bloodstream infection. During the last years *N. gonorrhoeae* has evolved into a superbug with resistance to previously and currently recommended antimicrobials for treatment of gonorrhoea^17^. Herein, we demonstrate that a synthetic short-chain ceramide analog showed antimicrobial activity against pathogenic *Neisseriae*, and preliminary data indicate that this analog caused dissipation of the membrane potential. Broth microdilution assays were performed to determine the minimum inhibitory concentration (MIC) and the minimum bactericidal concentration (MBC) of sphingolipids against both species. *Escherichia coli* and the Gram-positive bacterium *Staphylococcus aureus* were included as positive controls. In addition, the bactericidal activity against *N. meningitidis* was studied in time-killing assays. In this context, we aimed to analyse the mechanism of lipid antimicrobial activity and applied “click-chemistry” to ceramides, which were equipped with an azide group in the acyl-side chain^18^. The influence of substitution of one hydroxyl group by an azide on the antimicrobial activity was assessed and modified ceramide analogs were used to study uptake and localization of functionalized ceramide analogs within the *N. meningitidis* membrane by confocal laser scanning (CLSM) and at subdiffraction resolution by *direct* stochastic optical reconstruction microscopy (*d*STORM)^19,20^.

## Results

Previous studies have demonstrated that numerous sphingoid bases and fatty acids act as antibacterial agents against a variety of Gram-positive and Gram-negative bacteria^6^. Among these is the long chain base sphingosine^7^. We first estimated MIC and MBC values for sphingosine for the Gram-negative organism *N. meningitidis* and the closely related species *N. gonorrhoeae* by broth microdilution assays. MIC, MIC_50_ and MBC values of 4 µg/ml, 1.97 µg/ml and 4 µg/ml, respectively, for *N. meningitidis* MC58 and 4 µg/ml (MIC), 1.25 µg/ml (MIC_50_) and 8 µg/ml (MBC) for *N. gonorrhoeae* FA1090 were determined (Table 1). *E. coli* and the Gram-positive organism *S. aureus* were included as control organisms and MIC, MIC_50_ and MBC values of 16 µg/ml, 8.39 µg/ml and 16 µg/ml and 8 µg/ml, 1.22 µg/ml and >64 µg/ml, respectively, were estimated (Table 1). MIC_50_ values of 8.39 µg/ml and 1.22 µg/ml are in agreement with recently published data^6^.

**Table 1:** MICs and MBCs of sphingosine, short-chain C_6_-ceramide, long-chain C_16_-ceramide and C_6_/C_16_-ceramide analogs against *N. meningitidis, N. gonorrhoeae, E. coli and S. aureus*.

Compound	*N. meningitidis* (MC58)			*N. gonorrhoeae* (FA1090)		
	^a*^MIC	^**^MIC_>50_	^#^MBC	^*^MIC	^**^MIC_>50_	^#^MBC
sphingosine	4	1.97	4	4	1.25	8
C_6_-ceramide	2	0.69	64	1	0.18	>64
α-C_6_-ceramide	>64	—	>64	>64	—	>64
ω-C_6_-ceramide	2	0.8	4	2	0.8	4
C_16_-ceramide	16	8.39	>64	>64	—	>64
α-C_16_-ceramide	64	16.4	64	32	21	64
ω-C_16_-ceramide	>64	—	>64	>64	—	>64
**Compound**	***E. coli*** **ATCC 25922**			***S. aureus*** **ATCC 29213**		
	^a*^ **MIC**	^**^ **MIC**_**>50**_	^**#**^ **MBC**	^*^ **MIC**	^**^ **MIC**_**>50**_	^**#**^ **MBC**
sphingosine	16	8.39	16	8	1.22	>64
C_6_-ceramide	>64	—	>64	>64	—	>64
α-C_6_-ceramide	>64	—	>64	>64	—	>64
ω-C_6_-ceramide	>64	—	>64	>64	—	>64
C_16_-ceramide	>64	—	>64	>64	—	>64
α-C_16_-ceramide	>64	—	>64	>64	—	>64
ω-C_16_-ceramide	>64	—	>64	>64	—	>64

^a^MIC and MBC (µg/ml)

^*^MIC is defined as the lowest concentration of lipid that reduced growth by more than 95%

^**^MIC_>50_ is defined as the lowest concentration of lipid that reduced growth by more than 50%

^#^MBC is defined as the lowest concentration of lipid that prevented growth.

We next tested the antibacterial activity of ceramides, including short-chain C_6_ and long-chain C_16_-ceramides, and estimated MIC and MBC values as before. Interestingly, while long-chain C_16_-ceramides were not active against *N. meningitidis* and *N. gonorrhoeae* (Table 1), short-chain C_6_-ceramides displayed significant antimicrobial activity against both species: MIC, MIC_50_ and MBC values of 2 µg/ml, 0.69 µg/ml and 64 µg/ml, respectively, for *N. meningitidis* MC58 and 1 µg/ml (MIC), 0.18 µg/ml (MIC_50_) and ≥64 µg/ml (MBC) for *N. gonorrhoeae* FA1090 were determined (Table 1).

We next made use of recently synthesized azido-functionalized ceramide analogs^21,22^ allowing bio-orthogonal click–reactions^18^ in order to follow uptake and transport of fluorescently labelled ceramides in *N*. *meningitidis*. Four different ceramide analogs were synthesized, including α–azido-C_6_-ceramide, ω–azido-C_6_-ceramide, α–azido-C_16_-ceramide and ω–azido-C_16_-ceramide (see Fig. 1) and first analysed for their antibacterial activity. Interestingly, when an additional azido group was added at the ω-position of the fatty acid chain of C_6_-ceramide, the antibacterial activity was even increased: the ω–azido-C_6_-ceramide analog displayed a MIC value of 2 µg/ml, MIC_50_ value of 0.8 µg/ml and MBC value of 4 µg/ml for *N. meningitidis* and a MIC value of 2 µg/ml, MIC_50_ value of 0.8 µg/ml and MBC value of 4 µg/ml for *N. gonorrhoeae* (Table 1). In contrast, modification at the α-position of the fatty acid chain of C_6_-ceramide showed no significant activity against *N. meningitidis* (MIC and MBC value ≥64 µg/ml) or against *N. gonorrhoeae* FA1090 (MIC and MBC values ≥64 µg/ml). For *E. coli* ATCC 25922 and *S. aureus* ATCC 29213 the short-chain C_6_-ceramide MIC/MBC values were ≥64 µg/ml, and antibacterial activity did not increase after modification of the fatty acid chain neither at the α- or ω-position (Table 1).

### Effect of media on MIC and MBCs

It is well known that the inoculum size, the type of growth medium, the incubation time and the inoculum preparation method can influence MIC values. Many fastidious bacterial species including *N. meningitidis* and *N gonorrhoeae* do not grow satisfactorily using standard *in vitro* susceptibility testing approaches with unsupplemented media. For example, standard clinical laboratory broths, e.g. brain hart infusion or cation-adjusted Mueller-Hinton broth, allow multiplication of meningococci and gonococci only from large inocula. Recently a chemically defined liquid medium (Graver-Wade medium (GW)) was described, which permits growth of *N. meningitidis* and *N. gonorrhoeae* from low inocula^23^. We therefore next tested the antibacterial activity of ceramides by broth microdilution assays using GW medium. As shown in Table 2 the MIC values of sphingosine, C_6_-ceramide and ω–azido-C_6_-ceramide in GW medium shifted within 1 to 2 dilutions of the MIC tested in proteose peptone medium as shown in Table 1.

**Table 2:** MICs and MBCs of sphingosine, short-chain C_6_-ceramide, long-chain C_16_-ceramide and C_6_/C_16_-ceramide analogs against *N. meningitidis* and *N. gonorrhoeae* determined in Graver-Wade medium.

Compound	*N. meningitidis* (MC58)			*N. gonorrhoeae* (FA1090)		
	^a*^MIC	^**^MIC_>50_	^#^MBC	^*^MIC	^**^MIC_>50_	^#^MBC
sphingosine	4	1.58	4	2	1.19	4
C_6_-ceramide	4	2.32	8	8	2.01	32
α-C_6_-ceramide	>64	—	>64	>64	—	>64
ω-C_6_-ceramide	4	2.03	>64	4	1.46	16
C_16_-ceramide	16	7.64	16	16	8.63	32
α-C_16_-ceramide	32	17.6	32	32	20.4	64
ω-C_16_-ceramide	>64	—	>64	>64	—	>64

^a^MIC and MBC (µg/ml)

^*^MIC is defined as the lowest concentration of lipid that reduced growth by more than 95%

^**^MIC_>50_ is defined as the lowest concentration of lipid that reduced growth by more than 50%

^#^MBC is defined as the lowest concentration of lipid that prevented growth.

### Time-killing studies

**Figure 2:**
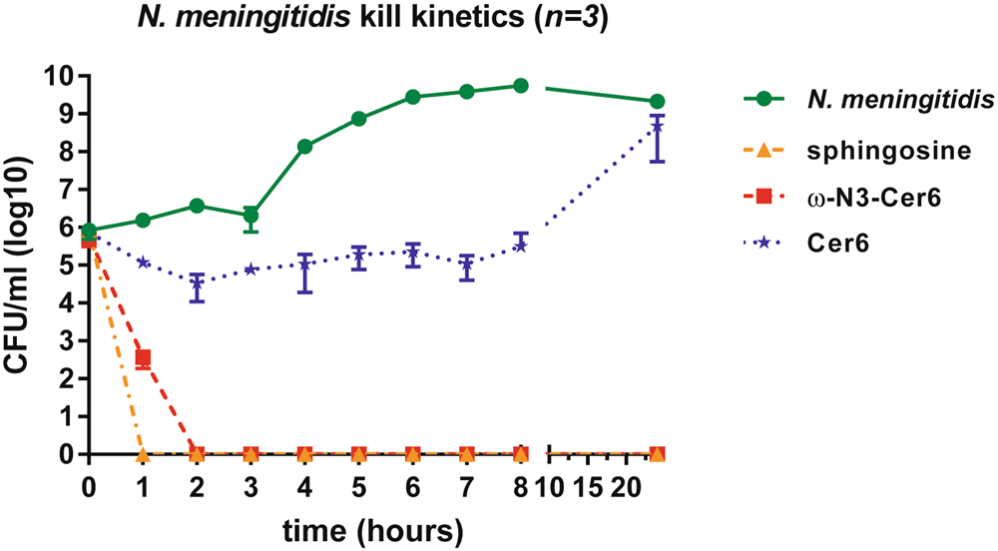
Kinetic killing of *N. meningitidis* with lipid treatment (sphingosine, unmodified C_6_-ceramide and ω-azido-C_6_-ceramide) at 1 time the MIC. Where no bacteria were recovered, +1 was added to the zero values before log transformation of the data. A geometric mean of *n* = 3 is shown for each data point. The error bars show mean ± SD of triplicate experiments.

Next, time-killing studies for sphingosine, the unmodified C_6_-ceramide from Avanti Polar and ω–azido-C_6_-ceramide were performed on *N. meningitidis*. Both sphingosine and ω–azido-C_6_-ceramide decreased the number of CFU/ml over the 0- to 24-h time period against *N. meningitidis* using 1 X MIC (Fig. 2). Kinetic assays showed that killing of *N. meningitidis* with sphingosine and ω–azido-C_6_-ceramide occurred within 1 h and 2 h, respectively (Fig. 2), whereas killing of *N. meningitidis* with the unmodified C_6_-ceramide was more gradual: *N. meningitidis* had at least a 1.3-log_10_ decrease in the number of CFU/ml with treatment with the unmodified C_6_-ceramide at 2 h post treatment, never showing a greater than 1.3 log reduction from the initial inoculum. Moreover, a slight degree of regrowth was seen for *N. meningitidis* at the 24-h time point for the unmodified C_6_-ceramide (Fig. 2). Treatment of the bacterial culture with ω–azido-C_6_-ceramide at 1 X MIC caused at least a 2.4-log_10_ reduction in the number of CFU/ml at 1 h and a 5.8-log_10_ decrease in the number of CFU/ml at 2 h. Thus, sphingosine and ω–azido-C6-ceramide demonstrated potent *in vitro* bactericidal activity against *N. meningitidis* at concentrations 1 x the MIC, while a bacteriostatic activity was observed for unmodified C_6_-ceramide against *N. meningitidis* at concentrations 1 x the MIC. Exact Kruskal-Wallis tests confirmed highly significant differences among non-treated and sphingosine (p < 0.0001) or ω–azido-C_6_-ceramide (p < 0.0001) treated *N. meningitidis* (Table 3). Comparison of the trapezoidal area under the curves (AUCs) also showed significant differences after treatment with sphingosine and ω–azido-C_6_-ceramide (Table 3).

**Table 3:** Comparison of the trapezoideal-AUC significance probabilities^a^.

	*N. meningitides* alone	Treatment with		
		C_6_-ceramide	sphingosine	ω–C_6_-ceramide
AUC	215.54	154.27	2.95	5.44
Trapezoidal-area significance probability^b,c^ (P value)		0.039792	<0.0001	<0.0001

^a^AUC for *N. meningitidis* was compared across lipid treatment as a summary measure of viability over the time course. AUC was calculated from time T_0h_ to the last sampling point (T_24h_). The significance probabilities are shown.

^b^Significance probability associated with the exact nonparametric Kruskal-Wallis test of the null hypothesis that the distribution of the trapezoidal area is the same across all treatment groups.

^c^Significance level 0.05.

### Toxicity testing

**Figure 3:**
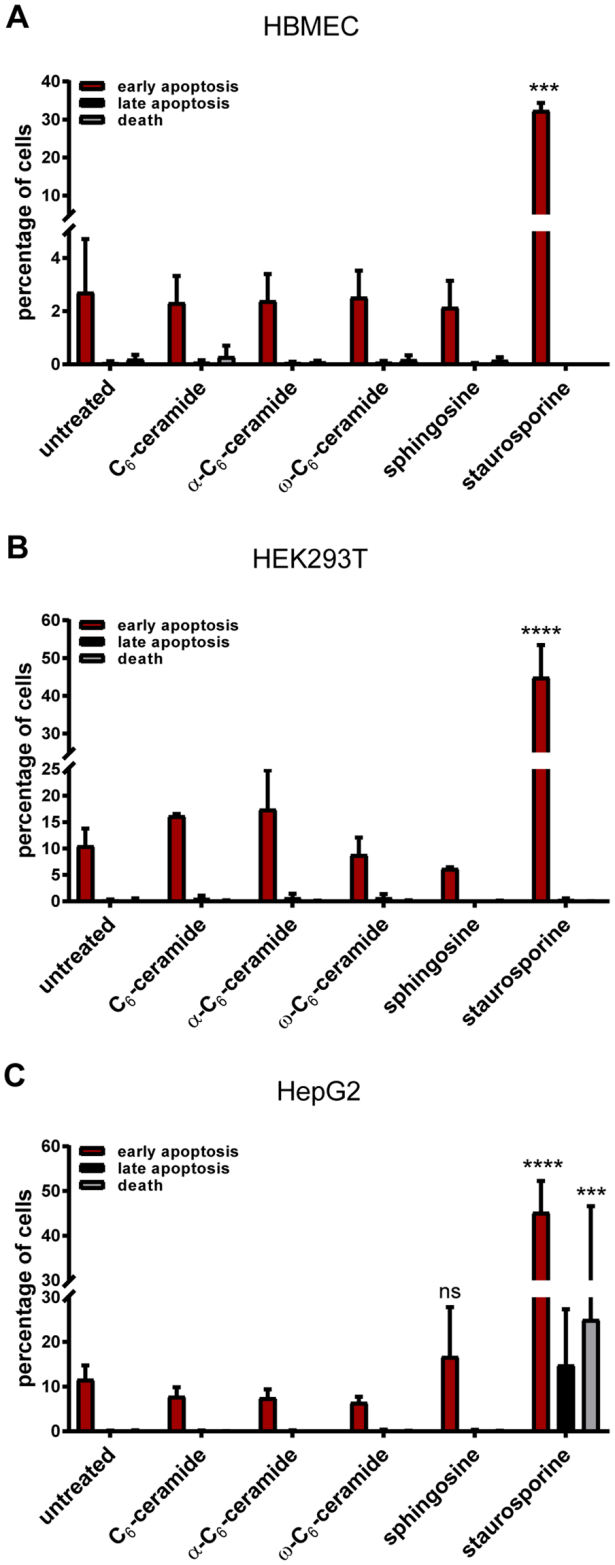
Effects of unmodified short-chain C_6_-ceramide (C_6_-ceramide), ω-azido-C_6_-ceramide, α-azido-C_6_-ceramide and sphingosine on (**A**) HBMEC, (**B**) HEK293T and (**C**) HepG2 cell apoptosis. Cells were left untreated or were treated with 5 µM of the compounds overnight. Staurosporine in a concentration of 1 µM was used as a positive control. Apoptosis was quantified using flow cytometry after staining with annexin V (AnnV)/PI. Percentage of early (AnnV^+^/PI^−^), late apoptotic cells (AnnV^+^/PI^+^) and dead cells (AnnV^−^/PI^+^) are shown. Data are presented as the mean ± SD of triplicate experiments. ***P < 0.001 in Students t-test relative to untreated cells.

We next tested the cytotoxicity induced by the unmodified and the functionalized ceramides in human brain microvascular endothelial cells (HBMEC), human embryonic kidney (HEK293T) cell line or the human hepatocellular carcinoma cell line HepG2 overnight using a concentration of 5 µM corresponding to the observed MIC values (2 µg/ml) determined for *N. meningitidis* and *N. gonorrhoeae*. Overnight treatment of the three different mammalian cells with staurosporine (1 µM) was used as a positive control. Notably, no significant cytotoxic effects neither on HBMEC, nor on HEK293T cells or HepG2 cells were observed for the concentration used in this study (Fig. 3).

### Uptake of functionalized ceramide analogs by bacteria

**Figure 4:**
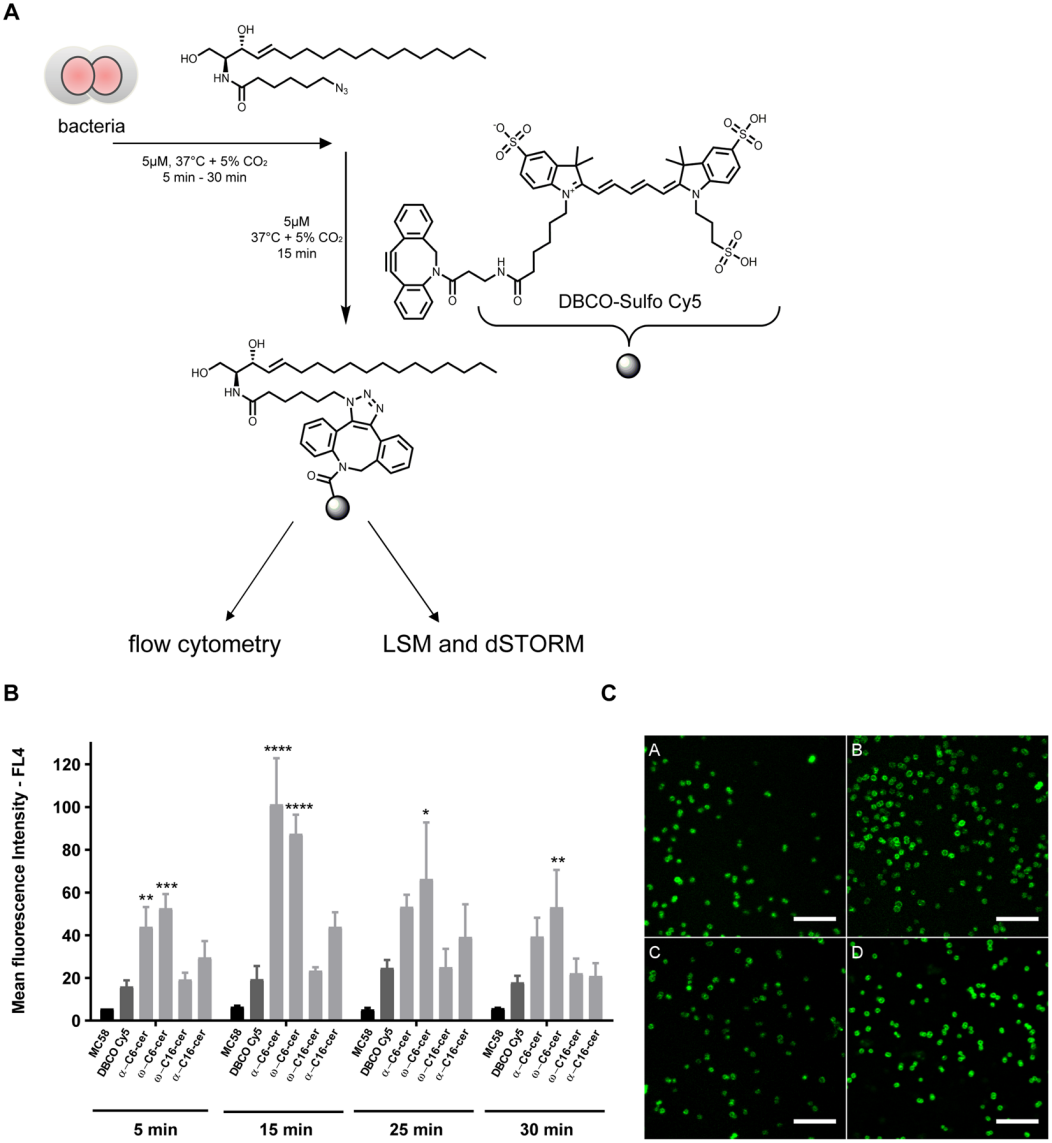
(**A**) Schematic of bacteria treated with ceramide analogs, followed by click reaction with DCBO-Sulfo-Cy5. (**B**) Azido-modified ceramides are rapidly incorporated into *N. meningitidis*. Bacteria were treated with 5 µM of α-azido-C_6_-ceramide (α-C6-cer), ω-azido-C_6_-ceramide (ω-C6-cer), α-azido-C_16_-ceramide (α-C16-cer) and ω-azido-C_16_-ceramide (ω-C16-cer), or DBCO-sulfo-Cy5 (DBCO Cy5) for indicated time points, washed, and ceramide incorporation levels were detected after 15 min DBCO-sulfo-Cy5-clicking and flow cytometry analysis. Three independent experiments with SD are shown. *, **, ***, ****P < 0.05, 0.01, 0.001, 0.0001 in one-way ANOVA and Tukey post hoc test relative to dye control (DCBO-sulfo-Cy5). (**C**) LSM images of *N. meningitidis* after incorporation of (**A**) α–azido-C_6_-ceramide, (**B**) ω–azido-C_6_-ceramide, (**C**) α–azido-C_16_-ceramide, and (**D**) ω–azido-C_16_-ceramide and clicking with DBCO-Sulfo-Cy5 by copper-free biorthogonal click chemistry. For visualization the contrast was adjusted for each image individually. Scale bar, 10 µm.

Little is known about the exact mechanism of lipid antimicrobial activity. In previous studies it has been shown that the bioorthogonal strain-promoted 3 + 2 cyloaddition between cyclooctyne and azide derivatives allow the conjugation between a biomolecule and a fluorescent dye^24–27^. In order to get insight into the mechanism we aimed at analysing the transport and localisation of the synthesized azido-functionalized ceramides in *N. meningitidis*. We therefore first established the incorporation kinetics of the functionalized ceramides in bacteria with different treatment times varying from 5 min to 30 min followed by fluorophore coupling. DBCO-Sulfo-Cy5 was selected as suitable dye for bio-orthogonal click reaction and the dye was coupled under copper-free conditions (Fig. 4A)^24^. Using flow cytometry analysis, we compared the uptake of α–azido-C_6_-, ω–azido-C_6_-, α–azido-C_16_- and ω–azido-C_16_-ceramide treated bacteria showing that both short-chain ceramide analogs efficiently accumulated in bacteria with a maximum peak at 15 min (Fig. 4B). Uptake levels of ω–azido-C_16_- and α–azido-C_16_-ceramide at 5 µM were substantially lower than that of the short-chain ceramide analogs. Flow cytometry data suggested that the long-chain ceramide analogs are less efficiently incorporated within this time interval. To confirm uptake levels, bacteria were treated with ceramide analogs as described above followed by 15 min fluorophore coupling and subsequent CLSM analyses. CLSM images of labelled bacteria confirmed that all clickable ceramides were efficiently incorporated into bacteria (Fig. 4C).

### Visualization of ceramide uptake in N. meningitidis (dSTORM)

**Figure 5:**
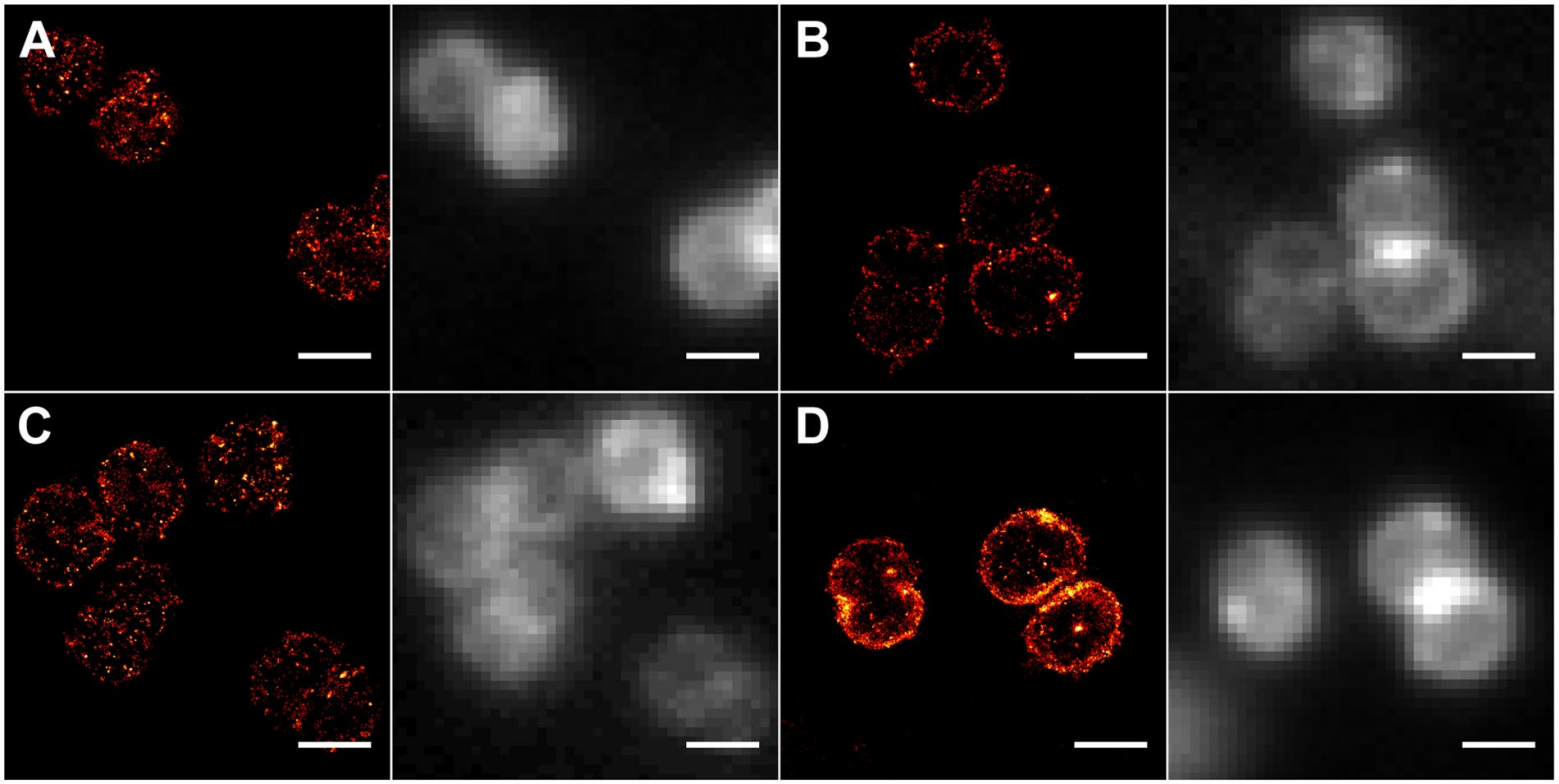
dSTORM and corresponding wide-field fluorescence of *N. meningitidis* after treatment with (**A**) α–azido-C_6_-ceramide, (**B**) ω–azido-C_6_-ceramide, (**C**) α–azido-C_16_-ceramide, and (**D**) ω–azido-C_16_-ceramide and clicking with DBCO-Sulfo-Cy5 by copper-free biorthogonal click chemistry. Scale bar, 1 µm.

In order to visualize the distribution of ceramides in *N. meningitidis* in more detail we used super-resolution imaging by *d*STORM, a single-molecule based localization microscopy technique that achieves virtually molecular resolution^28^ and has been already successfully used to image the distribution of clickable glycans and proteins in the plasma membrane of eukaryotes^24,29^. *d*STORM images clearly showed that all four ceramide analogs were incorporated into the bacterial membrane and the azide function was accessible for click reaction with DBCO-Sulfo-Cy5 (Fig. 5). None of the four analogs showed pronounced clustering. In contrast to the results of flow cytometry analysis, the super-resolution images demonstrated the strongest incorporation for the ω–azido-C_16_-ceramide. In addition, the ω–azido-C_16_-ceramide exhibited a tendency to accumulate in the contact areas between two bacteria. Control labelling experiments without ceramide treatment showed also non-specific staining of bacteria but at much lower efficiency (Fig. S1).

### Effect of sphingosine and functionalized ceramide analogs on bacterial cell physiology

In order to analyse the mechanism of the antibacterial activity of sphingosine and the functionalized ceramide analogs on bacteria, growing *S. aureus* strain ATCC 29213 or *N. meningitidis* strain MC58 were treated with diethyloxacarbocyanine, a validated indicator of the proton motive force in *S. aureus* and other bacteria^30^. This two-colour flow cytometry assay showed the expected shift in the red:green fluorescence ratio following addition of the proton ionophore carbonyl cyanide-m-chlorophenylhydrazone (CCCP) to the bacteria (Fig. S2): We observed a red:green ratio of about 6 for untreated stained *S. aureus*, which decreased to a red:green ratio of about 2 after incubation with CCCP. A similar decrease of the ratio was observed after treatment of *S. aureus* with sphingosine, indicating the collapse of the proton gradient (Fig. S2A). The red:green ratio for untreated stained *N. meningitidis* was about 1.8. The ratio also decreased significantly when bacteria were treated with sphingosine or ω–azido-C_6_-ceramide, whereas the red:green ratio was unaltered or even increased after treatment with α–azido-C_6_-ceramide (Fig. S2B and C). Taken together these preliminary findings indicate that both sphingosine and ω–azido-C_6_-ceramide dissipate the bacterial cell’s membrane potential in *N. meningitidis* and/or *S. aureus*.

## Discussion

Previous studies have demonstrated that numerous sphingolipids, extracted and purified from natural sources, can act as bactericidal agents against various microorganisms^4,6–10,31^. Sphingolipids showed growth inhibitory activity against Gram-positive and Gram-negative bacteria, fungi or microalgae^6–8,10,31^. The degree of the antibacterial activity depends on the sphingolipid structure and the microorganism tested. One of the best studied sphingolipids regarding antimicrobial activity is the long chain base sphingosine. Sphingosines form a primary part of sphingolipids, a class of cell membrane lipids that include sphingomyelin, an important phospholipid. Several studies have shown that sphingosine (and dihydrosphingosines and 6-hydroxysphingosines) are potent antimicrobials^4,6,7,14,32,33^. Of note, recent studies demonstrated that sphingosine is also an important first-line defence of healthy airways against *Pseudomonas aeruginosa* ^11^. Herein we demonstrated that sphingosine has also a significant antimicrobial activity against the Gram-negative species *N. meningitidis* and *N. gonorrhoeae*.

Ceramides are a structurally heterogeneous and complex group of sphingolipids containing derivatives of sphingosine bases in amide linkage with a variety of fatty acids. Differences in chain length, type and extent of hydroxylation, saturation etc. are responsible for the heterogeneity of sphingolipids. Some of the most structurally complex ceramides are found in the skin, which includes the presence of a very-long-chain fatty acid (C30 to C32) with an ω-hydroxyl group that is esterified to another fatty acid^34–36^, and in testis, which contains neutral glycosphingolipids with very-long-chain (C26 to C32) polyunsaturated fatty acids^37,38^. Ceramides with very short fatty acids (C2) have also been found in mammals and are formed by an acetyl transfer from platelet-activating factor to sphingenine^39,40^. In this study we now show that short-chain C_6_-ceramides and a functionalized ω–azido-C_6_-ceramide are active against *N. meningitidis* and the related species *N. gonorrhoeae*. Interestingly, they were inactive against *E. coli* and *S. aureus*. Time-killing kinetics revealed a significant decrease in mean viable count of *N. meningitidis* treated with sphingosine or ω–azido-C_6_-ceramide. *N. meningitidis* was efficiently killed by ω–azido-C_6_-ceramide within 2 h of treatment.

At present, little is known about the exact mechanism of lipid antimicrobial activity, although recently published findings suggest that sphingosine causes ultrastructural damage in *E. coli* and *S. aureus* ^31^. Antimicrobial lipids may penetrate and disrupt the cell wall layer of bacteria or may alter the cytoplasmic membrane. It is also likely that they might directly penetrate the cell wall and cytoplasmatic membrane and enter the cytoplasm. Herein, bacteria might accumulate them as intracellular inclusion. In order to get insight into the mechanism, we made use of azido-functionalized sphingolipids to visualize lipid uptake and localization in bacteria. We observed that the ceramide analogs are efficiently incorporated into the bacterial membrane within a short time period (Figs 4 and 5).

Recently, it has been shown by *d*STORM that ceramides are enriched in ceramide-rich platforms with a size of ~75 nm in the plasma membrane of eukaryotes^41^. In contrast, the ceramide distribution in *N. meningitidis* membranes appears mostly homogeneous. Only the long chain ceramide ω–azido-C_16_-ceramide accumulated slightly in contact zones to other bacteria (Fig. 5D). However, differences in the incorporation efficiency are difficult to quantify because α and ω-azido groups exhibit a different accessibility for click reaction and the different lengths of the alkyl side chains (C6 and C16) result in different incorporation efficiencies and geometries^22^. Moreover, probe preparation for flow cytometry differed from that for CLSM and *d*STORM. Bacteria were incubated for additional 30 min before analyzing by CLSM and/or *d*STORM to allow efficient binding of the bacteria to pre-coated chamber slides. It is likely that the additional incubation time probably resulted in damage of the cell wall when ω–azido-C_6_-ceramide was used thus resulting in less efficient staining and signal intensity detected by *d*STORM compared to treatment with the ω–azido-C_16_-ceramide analog.

However, localization studies alone do not allow to draw any conclusions about the mode of action of the substance, and a more systematic examination of the effect of sphingosine and short-chain ceramide-analogs on growth and membrane integrity of *N. meningitidis* is needed. In preliminary experiments, we found that treatment of bacteria with both sphingosine and ω–azido-C_16_-ceramide resulted in depolarization of the membrane potential (unpublished data): growing *S. aureus* and *N. meningitidis* strains were treated with diethyloxacarbocyanine, a validated indicator of the proton motive force in *S. aureus* and other bacteria. This two-color flow cytofluorometry assay showed the expected shift in the red-green fluorescence ratio following the addition of the protonophore carbonyl cyanide-m-chlorophenylhydrazone (CCCP) to growing cells. Treatment of *S. aureus* with sphingosine and of *N. meningitidis* with sphingosine or ω–azido-C_6_-ceramide yielded a color ratio shift similar to CCCP treatment (unpublished data), indicating the collapse of the proton gradient.

It is well known that the inoculum size, the type of growth medium, the incubation time and the inoculum preparation method can influence MIC values. Many fastidious bacterial species including *N. gonorrhoeae* and *N. meningitidis* do not grow satisfactorily using standard *in vitro* susceptibility testing approaches with unsupplemented media. Therefore, modifications have been made to the standard CLSI/EUCAST MIC methods to allow laboratories to perform reliable antimicrobial susceptibility testing. Such modifications typically involve the use of test media with supplemental nutrients, prolonged incubation times, and/or incubation in an atmosphere with an increased concentration of carbon dioxide. Herein, we either used proteose peptone medium or the recently described Graver-Wade^23^ medium to determine MIC values of sphingosine, ceramides and ceramide analogs. Interestingly, MIC and MBC values were influenced by the type of growth medium and values shifted within 1 to 2 dilutions. The difference might lie in the degree of free and protein-bound ceramides and sphingosine in the different test media solutions.

Infections caused by pathogenic *Neisseriae* are normally treated with antibiotics; however, the increasing occurrence of antibiotic-resistant bacterial strains as seen for *N. gonorrhoeae* ^17,42–44^ makes it highly desirable to identify new antimicrobials. Short-chain ceramides and their derivatives could prove useful in this regard, since they have high antibacterial activity and low toxicity at a bactericidal concentration. Here, we report for the first time the antimicrobial activity of synthetic ceramide analogs on *N. meningitidis* and the related species *N. gonorrhoeae*. Based on the results represented within this study a systematic functional analysis of the antimicrobial mechanism may enable the development of more efficient synthetic ceramide analogs with potential for therapeutic intervention. However, various hurdles and challenges will have to be overcome in the development of sphingolipid analogs or mimetics as therapeutic agents to treat a bacterial infections in particular avoiding interaction of the compounds with the host cells and preventing host cell cytotoxicity.

Synthetic ceramide analogs have been prepared for a wide range of purposes and are particularly of interest for the development as therapeutic agents in the treatment of cancer^45^. For example, ceramide analogs have been synthesized to explore structure-function relationships in cell signaling^46^, or as inhibitors of enzymes of ceramide metabolism^47^. Other analogs have shown activity as potential anticancer agents, such as L-threo-C6-pyridinium-ceramide-bromide^48^ or (2S,3R)-(4E,6E)-2-octanoyl-amidoocta-decadiene-1,3-diol (4,6-diene-Cer) with antiproliferative activity in breast cancer cells^49^. Unfortunately, ceramides are highly hydrophobic, poorly water-soluble molecules limiting their potential therapeutic utility. To overcome this hurdle, synthetic ceramide analogs have been developed that offer reduced lipophilicity or near complete water solubility. In recent years, there have been remarkable improvements in the design and delivery of ceramide analogs^45^. Intriguing new approaches have been the generation of water soluble pyridinium-ceramides^48,50^ or the delivery of pegylated liposomes^51^. In terms of systemic delivery of ceramide the advent of nanotechnology has shown the greatest promise^45^.

## Materials and Methods

### Bacterial strains, mutants and culture conditions

Isolate MC58 was used as a representative strain of the species *N. meningitidis. S*train MC58 is a serogroup (Sg) B strain of the sequence type (ST)-74 (ST-32 clonal complex [cc]), which was isolated in 1983 in the UK and was kindly provided by E. R. Moxon^52^. Isolate FA1090 was used as a representative strain of the species *N. gonorrhoeae*. Strain FA1090 is a porin serotype PIB-3, streptomycin (Sm)-resistant strain, originally isolated in the 1970s from the endocervix of a woman with disseminated gonococcal infection^53^ and has been used extensively in experimental human infection studies^54^. *Escherichia coli* ATCC 12759 and *S. aureus* isolates ATCC 29213 were included as control strains and to obtain information about Gram-negative and Gram-positive susceptibility and resistance. *N. meningitidis* strain MC58 and *N. gonorrhoeae* strain FA1090 were cultured on Columbia Agar with 5% sheep blood (COS; bioMérieux, Lyon, France) and incubated at 37 °C with 5% CO_2_ overnight. Liquid culturing was performed in proteose-peptone medium (PPM) plus 1% Kellogg’s supplement I and II (PPM^+^). *E. coli* ATCC 12759 and *S. aureus* ATCC 29213 were grown in Mueller-Hinton broth (Becton Dickinson, Maryland, USA) at 37 °C.

### Preparation of lipids

d-*erythro*-Sphingosine (C18) was obtained from Santa Cruz Biotechnology, Heidelberg, Germany. Unmodified C_6_-ceramide and C_16_-ceramide were obtained from Avanti Polar Lipids (Alabama, USA). Lipids were dissolved in ethanol, stored at −20 °C and protected from light. The azido ceramides (Fig. 1B) were synthesized as previously described^22^.

### Antimicrobial assay

Broth microdilution assays were used to determine the minimal inhibitory concentration (MIC), the MIC_50_, and the minimal bactericidal concentration (MBC) of D-*erythro*-sphingosine, short-chain ceramides or long-chain ceramides and their analogs. Briefly, lipid suspensions were diluted in PPM^+^, Graver-Wade medium (GW)^23^ or cation-supplemented Mueller-Hinton broth (for *E. coli* and *S. aureus*), which meets the requirements of the EUCAST standard, in standard 96 well microtiter plates (Sarstedt, Nuembrecht, Germany). Microdilution plates were prepared using serial twofold dilutions of the lipids (concentration ranging from 64 µg/ml to 0.0625 µg/ml in a total volume of 75 µl) in the respective media. At concentration higher than 64 µg/ml the lipids had an optical density that interfered with the determination of the MIC. To prepare the inoculum, all bacteria cell suspensions were adjusted to McFarland 0.5 (1–2 × 10^8^ CFU/mL). The suspension was further diluted to provide a final inoculum density of 5 × 10^5^ CFU/ml in the wells of the microdilution panels in a volume of 75 µl equal to the volume of diluted lipid. The plates were incubated for 16 h at 37 °C and 5% CO_2_ (for *N. meningitidis* and *N. gonorrhoeae*) and 37 °C (for *S. aureus* and *E. coli*). The optical density of bacterial growth was read at 540 nm in a spectrophotometer (Infinite F200 Pro Reader, Tecan Group, Maennedorf, Switzerland). The MIC was defined as the lowest concentration of an antibacterial agent that prevented visible growth under the test conditions, the MIC_50_ was defined as the lowest concentration of lipid that reduced growth by more than 50%, and the MBC was defined as the concentration of lipid that prevented growth. Quality control was monitored with *E. coli* ATCC 12759 and *S. aureus* isolates ATCC 29213.

### Killing kinetic assays

Killing kinetic experiments were performed according to previously published methods^55^. Briefly, freshly prepared colonies were resuspended in 10 ml PPM + medium and incubated in a shaker at 37 °C, 200 rpm for 1 to 2 h. Cultures were then diluted to a 0.5 McFarland standard and further diluted so that the starting inoculum was approximately 1 × 10^6^ CFU/ml. Sphingosine, Avanti Polar unmodified C_6_-ceramide and ω–azido-C_6_-ceramide was added to the prepared bacterial suspension so that the final concentration was 1 X the MIC of the compounds tested. A growth control with no lipid was also included. The starting inoculum was determined from the growth control tube immediately after dilution and was recorded as the bacterial CFU count at time zero. After addition of the compounds the tubes were incubated in a shaker at 37 °C, 200 rpm and viability counts were estimated at 1, 2, 3, 4, 5, 6, 7, 8, and 24 h by removing 1 mL of the culture, diluting as appropriate, and plating 100 µl on COS agar plates. COS agar plates were incubated at 37 °C with 5% CO_2_ overnight. Colonies were counted using a ProtoCOL colony counter (Synbiosis, Cambridge, UK) and the results were recorded as the number CFU/ml. A ≥ 3-log_10_ decrease in the number of CFU/ml was considered bactericidal.

### Membrane potential analysis and flow cytometry

Membrane potential was determined by flow cytometry using a BacLight™ bacterial membrane potential kit (Molecular probes). This test is based on a fluorescent membrane-potential indicator dye, 3,3′-diethyloxacarbocyanine iodide (DiOC_2_(3)), along with a proton ionophore carbonyl cyanide-m-chlorophenylhydrazone (CCCP). DiOC_2_(3) at low concentrations exhibits green fluorescence in all bacterial cells, but it becomes more concentrated in healthy cells that are maintaining a membrane potential, causing the dye to self-associate and the fluorescence emission to shift to red. *S. aureus* and *N. meningitidis* were grown to mid-logarithmic phase, and 1 × 10^6^ cells per ml were treated with ethanol (solvent), sphingosine, α–azido-C_16_-ceramide or ω–azido-C_6_-ceramide, or incubated in either the presence or absence of 5 µM CCCP (used as a depolarized control) followed by incubation with 30 µM DiOC_2_(3) for 30 min at 37 °C and then analyzed by flow cytometry. DiOC_2_(3) was excited with the 488 nm argon laser, and emission was detected as follows: green fluorescence was detected using a 530/30 filter, and red fluorescence was detected with a 585/42 filter. The ratio of red to green fluorescence intensity were calculated using mean fluorescence intensities following the manufacturer’s description.

### Mammalian cell line

The simian virus 40 large T antigen-transformed human brain microvascular endothelial cells (HBMEC) were cultured as previously described^56–58^. Briefly, HBMECs were cultured in RPMI-1640 medium (Gibco Life Technologies, Karlsruhe, Germany) supplemented with 10% fetal calf serum (FCS, Gibco Life Technologies), 10% Nu serum^®^ IV (Corning, NY, USA 10%; Becton Dickinson), 1% sodium pyruvate (1 mM), 1% L-glutamine (2 mM), 1% non-essential amino acids (all purchased from GE Healthcare, Little Chalfont, UK), 5 U ml^−1^ heparin (Biochrom, Berline, Germany) and 30 µg mL^−1^ endothelial cell growth supplement (ECGS, CellSystems, Troisdorf, Germany). Cultures were incubated in a humid atmosphere at 37 °C with 5% CO_2_. Cells between the 10th and 25th passages were used for apoptosis analysis. HBMEC were cultured in T25 flasks (Corning Costar Corporation, Cambridge, MA, USA). The embryonic kidney cell line HEK293T and the hepatocellular carcinoma cell line HepG2 were purchased from the American Type Culture Collection, ATCC^®^ CRL-3216^™^ and ATCC^®^ HB-8065^™^, respectively. HEK293T and HepG2 were routinely cultured in Dulbecco’s modified Eagle’s medium (DMEM) + GlutaMAX^™^-I (Gibco Life Technologies, Karlsruhe, Germany) supplemented with 10% FCS.

### Apoptosis analysis

HBMEC, HEK293T or HepG2 cells were seeded in 6-well tissue culture plates to a density of 2 × 10^6^ cells/well. The medium was changed and cells were treated with the compounds overnight. Cells were harvested and washed once with 1 x PBS^−/−^ and once with Annexin V binding buffer (BD Biosciences). Cells were resuspended and transferred into a 500 μL siliconized polypropylene tube. Annexin V-Alexa Fluor 488 (Molecular Probes) was added at a 1:20 dilution and cells were incubated for 15 min at RT. Following this, propidium iodide (PI) was added at a final concentration of 1 μg/mL and cells were stained for a further 15 min at RT. After staining, cells were washed twice with PBS^−/−^ and fixed with 2% formaldehyde for 10 min on ice. Following fixation, cells were washed twice with PBS^−/−^ and treated with 50 μg/mL DNase-free RNase (Sigma, R4642) for 15 min at 37 °C. Cells were then washed once with PBS^−/−^ and immediately analyzed using a BD FACSCaliburTM flow cytometer (BD Biosciences) and BD CellQuestTM Pro Software (BD Biosciences). For each measurement, at least 10,000 cells were counted. Cells that stained positive for annexin V represented cells with intact membranes and externalized phosphatidylserine (early apoptosis) and cells positive for annexinV/PI represent cells that had lost membrane integrity (late apoptosis/necrosis). A modified Annexin V/ PI method was used to assess cell death^59^.

### Flow cytometry and click chemistry reaction

For detection of ceramide incorporation bacteria were fed with functionalized (α–azido-C_6_-ceramide, ω–azido-C_6_-ceramide, α–azido-C_16_-ceramide and ω–azido-C_16_-ceramide) ceramides at a concentration of 5 µM for indicated time points at 37 °C and 5% CO_2_, washed one time with 1 mL PBS and bacteria were exposed to 5 µM DBCO-Sulfo-Cy5 dye (Jena Bioscience, Jena, Germany) and the click reaction was performed for 15 min at 37 °C and 5% CO_2_. Bacteria were washed three times with 1 mL PBS, then resuspended in 500 µL FACS buffer (PBS + 5% FCS) and used for flow cytometry analysis.

### LSM and *d*STORM

LSM and dSTORM imaging was performed as previously described^41^. Briefly, bacteria were fed with functionalized ceramide for 15 min as described above, followed by a 15 min click chemistry reaction. Bacteria were washed three times with 1 mL PBS, resuspended in 100 µL RPMI-1640 medium and seeded on precoated chamber slides (Nunc™ Lab-Tek™ II Chamber Slide™, ThermoScientifc Fisher) for 30 min at 37 °C and 5% CO_2_. Bacteria were fixed with 2% PFA for 15 min at RT and chamber slides were stored overnight at 4 °C before microscopy experiment. For super-resolution microscopy bacteria were covered with switching buffer consisting of 100 mM ß-mercaptoethylamine (MEA, Sigma) in PBS and pH adjusted with potassium hydroxide to 7.4. Super-resolution measurements were performed at an inverted wide-field fluorescence microscope (IX-71; Olympus) with an oil-immersion objective (60x, NA 1.45; Olympus) and suitable excitation and emission filters (ZT405/514/635rpc; Chroma; EM01-R442/514/647-25; Semrock). The organic fluorophores (DBCO-Sulfo-Cy5) were excited with a 640 nm laser (Cube 640–100 C; Coherent). After passing a long- and bandpass filter (LP635; Semrock; HC 679/41; Semrock) photons were detected by an electron-multiplying CCD camera (iXon Ultra DU-897; Andor). The sample was homogenously illuminated with an irradiation intensity of ~ 7 kW/cm^2^ and super-resolved images were reconstructed with rapidSTORM 3.3.1^60,61^ from 30,000 frames with an exposure time of 20 ms. LSM was performed with a LSM700 (Zeiss, Germany) equipped with a Plan-Apochromat 63 × 1.4 oil-immersion objective and the PBS-covered sample was excited with a 639 nm solid state laser.

### Statistical analysis

Statistical analysis was performed using unpaired Student *t* test with ****p < 0.0001, ***p < 0.001, **p < 0.01, *p < 0.05. The exact Kruskal-Wallis test was performed to detect differences in killing kinetics, utilizing a 5% level of statistical significance. This nonparametric analog to analysis of variance (ANOVA) was used due to modest sample size and violation of the normality assumptions for parametric procedures. Moreover, the trapezoidal area under the curve (AUC) was determined to summarize the measurement of bacterial viability of treatment time course.

## Electronic supplementary material

Supplementary Information
