# Supplementary material for: Antibacterial activity of ceramide and ceramide analogs against pathogenic Neisseria
Source: Sci Rep. 2017 Dec 15;7:17627. doi: 10.1038/s41598-017-18071-w (PMC5732201; doi:10.1038/s41598-017-18071-w)
Supplement: Supplementary file 1 — Supplementary Information [file 41598_2017_18071_MOESM1_ESM.doc]

**Supplementary Information**

Antibacterial activity of ceramide and ceramide analogs against

pathogenic *Neisseria*

Jérôme Becam**^1^**, Tim Walter**^2^,** Anne Burgert**^3^**, Jan Schlegel**^3^**, Markus Sauer**^3^**, Jürgen Seibel**^2^** and Alexandra Schubert-Unkmeir**^1*^**

^1^Institute of Hygiene and Microbiology, Julius-Maximilian University Wuerzburg, Wuerzburg, Germany

^2^Institute for Organic Chemistry, Julius-Maximilian University Wuerzburg, Wuerzburg, Germany.

^3^Department of Biotechnology and Biophysics, Julius-Maximilian University Wuerzburg, Wuerzburg, Germany

*corresponding author

Mailing address:

Institute of Hygiene and Microbiology, University of Wuerzburg, Josef-Schneider-Straße 2

97080 Würzburg, Germany

Tel.: 0049 931 3146721; Fax: 0049 931 3146445

email: [aunkmeir@hygiene.uni-wuerzburg.de](mailto:aunkmeir@hygiene.uni-wuerzburg.de)


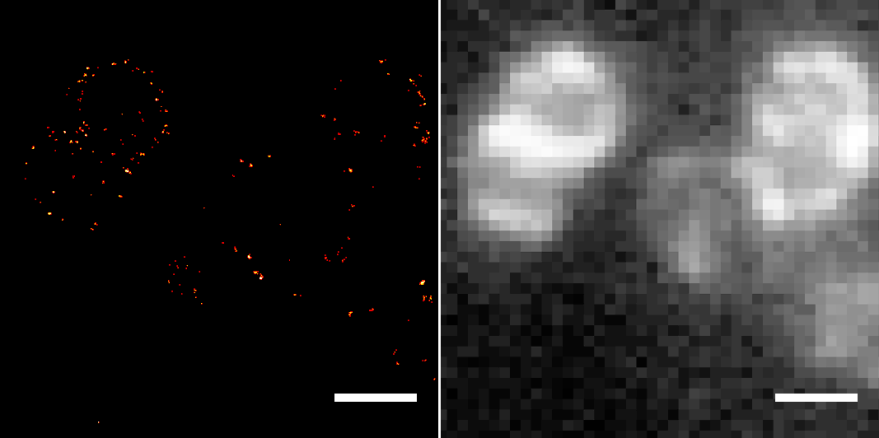


**Supplementary Fig. S1.** *d*STORM and corresponding wide-field fluorescence of *N. meningitidis* after clicking with DBCO-Sulfo-Cy5 by copper-free biorthogonal click chemistry . Scale bar, 1 µm.


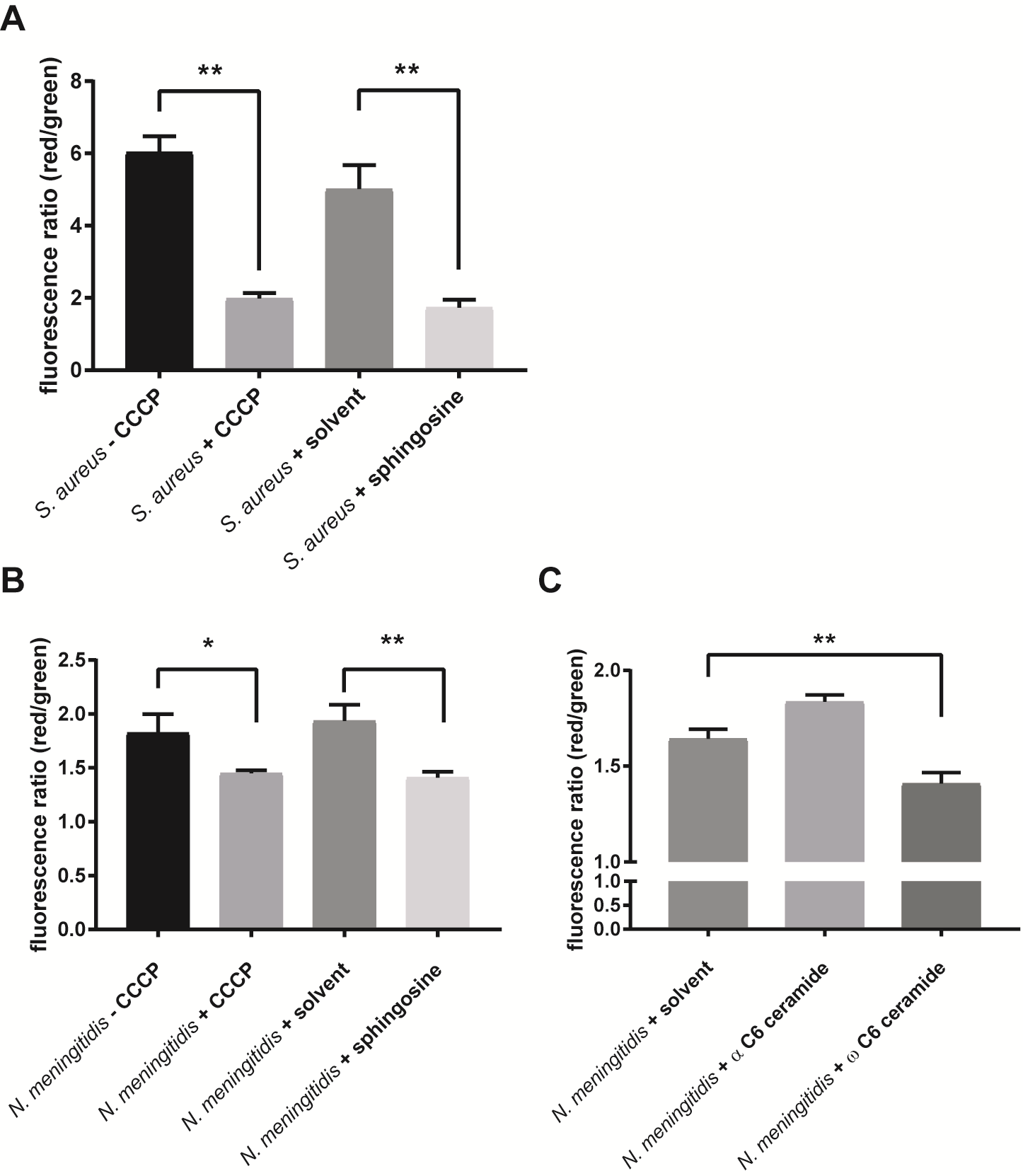


**Supplementary Fig. S2.** Detection of membrane depolarisation in *S. aureus* (A) and *N. meningitidis* (B, C) by sphingosine or ω–azido-C_6_-ceramide. *S. aureus* strain ATCC 29213 and *N. meningitidis* strain MC58 were incubated with DiOC_2_(3) (30 µM) for 30 min in either the presence or absence of CCCP (5 µM) or were treated either with ethanol (solvent), sphingosine (8 µg/ml for *S. aureus*, 4 µg/ml for *N. meningitidis*), α–azido-C_6_-ceramide (4 µg/ml), and ω–azido-C_6_-ceramide (4 µg/ml) and subjected to dual-color flow cytometry analysis. Red:green ratios were calculated using population mean fluorescence intensities as described in materials and methods. *, **: *P* < 0.05, 0.01 in Students t-test relative to control (- CCCP or solvent).
